# No Flies

**Published:** 1902-08-16

**Authors:** 


### The Hospital

A Journal of

The Medical Sciences and Hospital Administration.

Vol. XXXII.—No. 829. Edited by Sir Henry Burdett, K.C.B., and Solomon C. Smith, M.D., M.R.C.P. [August 16, 1902.

### "No Flies."

The scrap of slang which we have ventured to place at the head of this article has received the imprimatur of the great Oxford Dictionary, which gives, as one of the legitimate meanings of "fly," a trick, a dodge; so that it is now fully entitled to make its bow in the best of company. It has been recalled to our recollection by the reports of a very admirable discourse, lately delivered by Sir James Crichton Browne at the annual meeting of the Sanitary Inspectors' Association, in which, after describing with much fulness the part taken by the mosquito in the communication of malarial fevers, he went on to point out that other insects, long regarded as harmless, were beginning to fall under grievous suspicions, and to give prominence as an illustration to the *musca domestica*, the "busy, thirsty, curious fly," which, even in this country, in certain states of atmosphere and weather, is apt to be a nuisance, while in hot climates, and in the conditions engendered by camp life, it may often become a well-nigh intolerable and possibly also a dangerous one. In the Crimea, during the war, they were present in countless swarms, and would even alight in numbers upon a morsel of food between the plate and the mouth, so that it was hardly possible always to avoid swallowing them. In South Africa as Sir James Crichton Browne reminded his hearers, they were similarly abundant, and he quoted Dr. Tooth as saying that "they were all over our food, and the roofs of our tents were at times black with them." According to Sir William Church "they congregated specially round typhoid stools, and, on entering a hospital tent, it was possible at once to identify the typhoid patients by the grim pall of flies that had settled on them." Notwithstanding our cordial support of the water-boiling proposals of Dr. Leigh Canney, we have always entertained the conviction that flies, as well as water, must be reckoned among the probable channels for the diffusion of typhoid; and this evidence of their continued migration from the stools or the patient to the food of the sound man affords the strongest possible confirmation of such an opinion. Under ordinary conditions, it must be presumed that the rate of their multiplication, like that of other creatures, must be largely dependant upon the abundance or deficiency of food; but Sir James Crichton Browne told his hearers that a single female fly was capable of being the ancestress of twenty-five millions of descendants in the course of a single season; so that abundant provision has been made by Nature for their presence wherever their sustenance may be plentiful. In such circumstances it is almost manifest that the presence and activity of numerous flies affords at least strong presumptive evidence of the presence also of abundant dirt or refuse, and that the insects may therefore be regarded as affording an index or scale of measurement of what are comprehensively described as insanitary conditions. We fear it cannot be maintained that their utility as scavengers bears any proportion to the mischief, oftentimes, no doubt, the utterly unsuspected mischief, which they may do by contaminating food with diseased secretions containing specific bacteria; and Sir James was fully justified in directing the attention of his hearers to this contingency, and in pointing out to them that, although he did not know of any possibility that the proboscis of the fly, like that of the mosquito, might become a tube for the insertion of poison, yet he did know, from personal observation, that particles of matter of various kinds, encountered in his unclean wanderings, might adhere to the *tarsi* of his legs, to his *plantulæ* and *pulvilli*, and might thus be conveyed from point to point, and deposited upon materials intended for human food, especially upon cooked food which was liable to be consumed without further exposure to heat. It may fairly be concluded, that Sir James was correct not only in his general view of the question, and in his warning to sanitary inspectors that an unusual abundance of flies in the localities which they were called upon to visit should be held to justify suspicion, and to demand a search for the generally noxious refuse on which the creatures chiefly feed, but also in saying that the scarcity or absence of flies in a dwelling should be regarded as evidence of good housewifery. Other precautions to the value of which Sir James also called attention were that larders should be placed as remote as possible from dustbins or other sources of probable contamination, that they should be well ventilated through openings guarded by wire gauze, and that wire-woven safes and covers for the protection of stored provisions should be systematically used. There is a widely spread belief, which is at least a harmless one, to the effect that houses covered by Virginia creeper are very little visited by flies, the insects being either repelled by the plant or being so attracted by it as to remain among its foliage. We have seen more than one instance of a house so protected in which flies were conspicuous by their absence; but in all of them the houses themselves were unusually clean, and the problem of cause and effect might therefore admit of more than one hypothetical solution.

